# SARS-CoV-2, immunosenescence and inflammaging: partners in the COVID-19 crime

**DOI:** 10.18632/aging.103989

**Published:** 2020-09-29

**Authors:** Renato Domingues, Alice Lippi, Cristian Setz, Tiago F. Outeiro, Anita Krisko

**Affiliations:** 1 Department of Experimental Neurodegeneration, Center for Biostructural Imaging of Neurodegeneration, University Medical Center Goettingen, Goettingen, Germany; 2 Center of Excellence for Science and Technology-Integration of Mediterranean Region (STIM), Faculty of Science, University of Split, Split, Croatia; 3 Max Planck Institute for Experimental Medicine, Goettingen, Germany; 4 Translational and Clinical Research Institute, Faculty of Medical Sciences, Newcastle University, Framlington Place, Newcastle Upon Tyne, UK; 5 Department of Otolaryngology-Head and Neck Surgery, University Medical Center Goettingen, Goettingen, Germany

**Keywords:** aging, SARS-CoV-2, COVID-19, neuroinflammation, inflammaging, immunosenescence

## Abstract

Pneumonia outbreak in the city of Wuhan, China, prompted the finding of a novel strain of severe acute respiratory syndrome virus (SARS-CoV-2). Here, we discuss potential long-term consequences of SARS-CoV-2 infection, and its possibility to cause permanent damage to the immune system and the central nervous system. Advanced chronological age is one of the main risk factors for the adverse outcomes of COVID-19, presumably due to immunosenescence and chronic low-grade inflammation, both characteristic of the elderly. The combination of viral infection and chronic inflammation in advanced chronological age might cause multiple detrimental unforeseen consequences for the predisposition and severity of neurodegenerative diseases and needs to be considered so that we can be prepared to deal with future outcomes of the ongoing pandemic.

## INTRODUCTION

At the end of 2019, the novel severe acute respiratory syndrome coronavirus (SARS-CoV-2) emerged in Wuhan, China, as the causative agent of Coronavirus Disease 2019 (COVID-19) [1, 2]. The most prominent clinical symptom of COVID-19 is extensive lung damage, accompanied by respiratory distress of varying severity [3]. Within only 2-3 months, SARS-CoV-2 caused a worldwide health emergency and a pandemic, by infecting over 15 million people and, at the point of writing of this text, taking more than 633,000 lives. Within this short period, the pandemic has also triggered an avalanche of social and economic consequences that promise to continue growing, and that will scar our society [1].

SARS-CoV-2 belongs to the family of coronaviruses (CoV), together with SARS-CoV and Middle East respiratory syndrome CoV – two highly pathogenic viral strains that caused significant medical turmoil in the recent past and were responsible for considerable lethality [4]. The same family also includes several harmless viruses (HKU, 229E) [5]. The coronavirus family shares some overall similarities with the influenza A virus (IAV) H1N1 in the context of immune system activation, which includes allowing interferon-stimulated genes (ISG) effector response, responsible for the first defense against viral infection [6].

**Figure 1:**
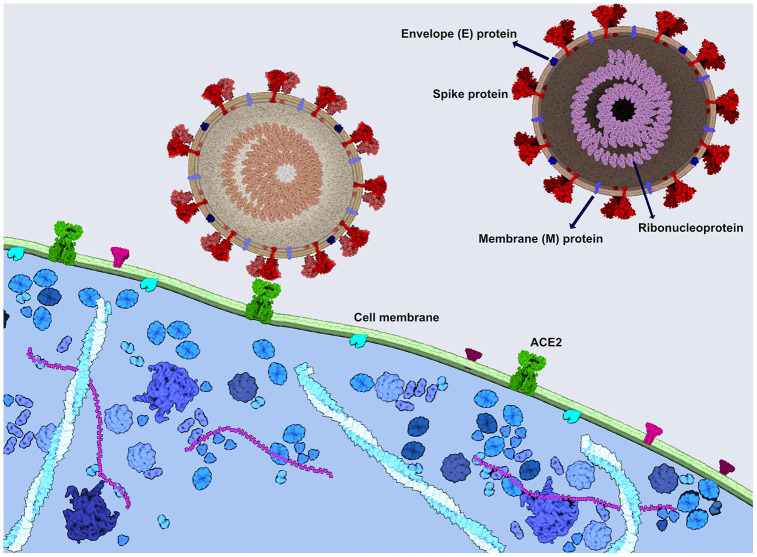
**SARS-CoV-2 spike protein binds to the ACE2 receptor to enter the cells.** Viral spike protein binds to the ACE2 receptor in the human cell membrane, followed by the internalization of the virus. SARS-CoV-2 consists also of the ribonucleoprotein, envelope protein and a membrane protein. The image was generated using CellPAINT Software [100].

SARS-CoV-2 is a large and enveloped virus with positive-sense, single-stranded RNA genome [7]. The infection is initiated by the binding of the viral spike (S) protein to ACE2 receptor at the host cell surface (Figure 1) [8], followed by the internalization and replication of the virus, culminating in the cell lysis and the exit of newly formed viral particles [9].

Although no treatment or preventive measures against SARS-CoV-2 exist at the present moment, the scientific community is working tirelessly, producing daily results on the molecular properties of the new virus and the plethora of its interaction with the host cells and tissues.

While at the clinical level, the respiratory problems are one of the main hallmarks of the disease, the molecular alterations among the severe cases of COVID-19 include signs of hyperinflammation characteristic of immunopathologies. The most striking example is a systemic inflammatory response known as cytokine release syndrome (or cytokine storm) due to massive T cell stimulation [10].

Here, we address the major clinical features of COVID-19 and discuss its potential effects on the aged population, from the perspective of its incidence and severity, as well as long-term effects in developing age-related diseases of the central nervous system.

On the one hand, aging affects the severity of COVID-19 and, on the other, is the leading risk factor for the development of neurodegenerative diseases [11]. Although the link between the SARS-CoV-2 and neurodegeneration has yet to be established, the cocktail of infection stress, chronic inflammation, and advanced chronological age may cause multiple detrimental unforeseen consequences to the risk and severity of neurodegenerative diseases. Therefore, it needs to be seriously considered so that we can be prepared to deal with future outcomes of the ongoing pandemic.

### Clinical aspects of SARS-CoV-2 infection

The clinical spectrum associated with SARS-CoV-2 infection varies among the infected population depending on the time point of the diagnosis. At the moment of seeking medical attention, the most common symptoms are fever (>37.4°C), fatigue, dry cough, myalgia, and dyspnea [12]. The reduced ability to smell, or hyposmia, has been characterized as a major symptom in otherwise mild cases [13]. The other typical symptoms associated with a common viral upper respiratory infection, such as nasal congestion and rhinorrhea, are very uncommon (< 5%) [14, 15].

The SARS-CoV-2 infection primarily affects adults, with fewer cases reported in children of 15 years or younger [15, 16]. The virus enters the host through the upper airway, and the viral load peaks at approximately day ten after the onset of symptoms [17]. The highest spread during the initial phase of the epidemic in Wuhan was observed as a human-to-human transmission among otolaryngologists [18].^.^Subsequent studies conducted on infected patients demonstrated high SARS-CoV-2 titers in the mucosa of the nasal and oral cavity [19], which represents the way SARS-CoV-2 enters the host, most readily transmitted by respiratory droplets and direct contact. The asymptomatic form of transmission may have contributed to the rapid spread of the disease [12], but there is still no scientific consensus regarding this mechanism [20–22].

A significant portion of patients infected with SARS-CoV-2 also shows neurological symptoms such as headache, nausea, and vomiting (<5%). Other described neurologic manifestations associated with SARS-CoV-2 infections are impaired consciousness and cerebrovascular disease [15, 23]. The first case of meningitis/encephalitis associated with SARS-CoV-2 infection was also recently reported [24].

SARS-CoV, a closely related virus, enters into human host cells mediated mainly by the angiotensin-converting enzyme 2 (ACE2) receptor, expressed in human airway epithelia and lung parenchyma, but also present in vascular endothelial cells, kidney cells, cells from the small intestine, and the brain (Figure 1) [25, 26]. Usually located on type I and II alveolar cells in the lung, the ACE2 receptor was also found to bind SARS-CoV-2 with an estimated binding affinity 10-20 times greater than the one of SARS-CoV [27]. The mechanism of entry into the host target cells, for both SARS-CoV and SARS-CoV-2, is warranted by the spike (S) protein [28, 29]. When attached to ACE2, the cellular transmembrane serine protease 2 (TMPRSS2) primes the spike protein to trigger the entry of the virus into the cell [19, 29]. Therefore, the spread of SARS-CoV-2 also depends on TMPRSS2 activity [29].

Neurotropism highlights the prerequisite of awareness towards SARS-CoV-2 entering the central nervous system. The neuroinvasive propensity of CoV has been documented for almost all of the β-CoV, including SARS-CoV [30], MERS-CoV [31], HCoV-229E [32] and HCoV-OC43 [23]. Evidence suggests that the virus might first invade peripheral nerve terminals, thus gaining access to the central nervous system via synapse-connected route [33, 34].

### SARS-CoV-2: immunosenescence and increased severity among older adults

Epidemiological studies show that older adults are the most affected by this pandemic [35], rendering the chronological age a risk factor in COVID-19. Moreover, studies reveal the variable host resistance between patients from the same age groups.

Casualties in all age groups are also associated with pre-existing conditions such as reduced lung function, cardiovascular problems, and oncological disease spectrum. However, other factors might affect the outcome of patients with COVID-19 [36], such as variable genetic background and epigenetic predisposition. All these effectors converge at the level of immune system attenuation.

Since the beginning of the SARS-CoV-2 outbreak, parallels were made with the influenza A virus H1N1 infection, due to its contributions to the mortality of the elderly. Influenza remains a serious global health threat that impacts all countries, with 290,000-750,000 influenza-related respiratory deaths worldwide every year [37].

Senescence defines a stable growth arrest induced when cells reach the end of their replicative potential or are exposed to various stressors, such as infection. Senescent cells accumulate in aging tissues and contribute to the development of age-related disorders [38]. However, it was only in 2011 when evidence was presented showing that the clearance of senescent cells can delay aging-associated diseases [39]. This discovery confirmed senescence as a hallmark of aging.

Like other tissues, the immune system is characterized by the decline of its functions with age (immunosenescence), reflected not only in increased cancer prevalence, autoimmune and other chronic diseases but also in greater susceptibility to infections [40]. Understood as a gradual deterioration of the immune system brought on by natural age advancement, immunosenescence originates as a disability of T Cells (CD4 as well as CD8 positive) to function correctly [41].

Senescence compromises the ability of CD4+ T cells to correctly activate, differentiate, proliferate, and respond to the H1N1 virus [42]. Aged CD4+ T cells accumulate intrinsic defects that contribute to a reduced helper function during influenza infection [43, 44]. In vivo studies conducted on senescent mice have evidenced low H1N1 influenza-specific antibody titers after influenza infection that reflects the age-related lowered immune response [44].

Viral infections are also known as stressors that can induce senescence in different cell lines. The Dengue virus can cause senescence in endothelial cells [45], and the Measles virus leads to cellular senescence in normal and cancer fibroblasts [46]. Senescent cells can play a role during viral infection by limiting the proliferation of damaged cells. In fact, these cells help to control the viral replication, while in experimental studies, senescence induction restricts the infection in mice [47]. Moreover, the NS1 protein of the avian influenza H7N9 virus can induce growth arrest and cellular senescence in Neuro2a cells [48]. Neurons infected with influenza A virus can respond to the infection by producing oxygen radicals and nitric oxide (NO) [49]. NS1 protein leads to an increased release of NO in Neuro2a cells which causes a reduced proliferation, enlarged cell morphology, an up-regulation of IL-6 and IL-8 as well as increased SA-β-gal activity, all features of senescent cells [48].

Immunosenescence offers insights into the differential resistance of young vs. old individuals, as well as men vs. women, to SARS-CoV-2 infection [50]. The depletion of B lymphocyte-driven acquired immunity is a characteristic of old age, affecting predominantly men [51]. Aging diminishes the upregulation molecules essential for T cell priming and also reduces antiviral interferon (IFN) production by alveolar macrophages and dendritic cells (DCs) [52].

**Figure 2:**
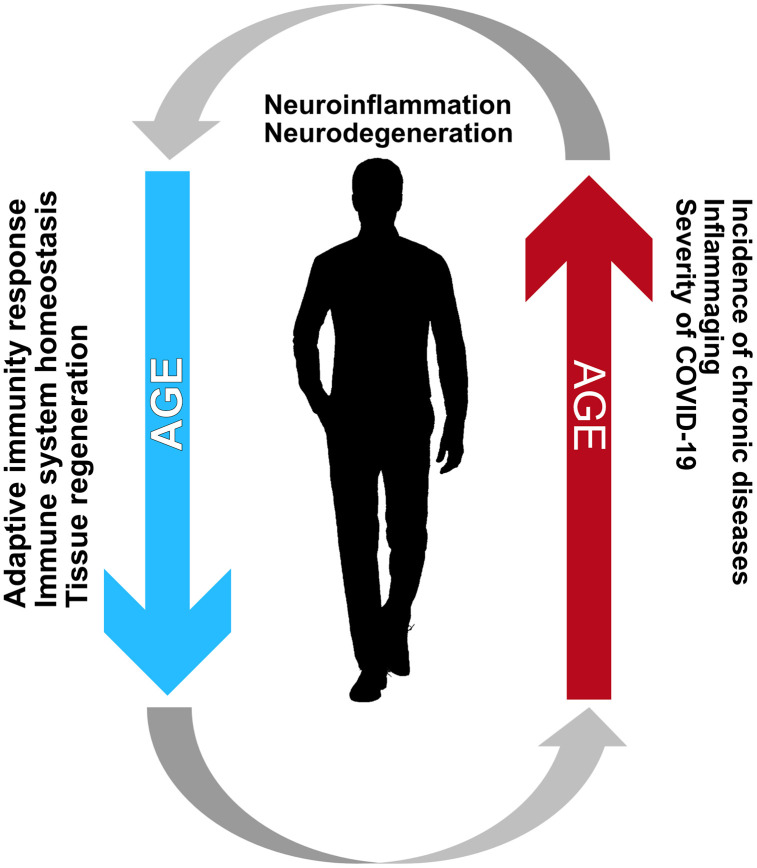
**Immunosencescence and inflammaging create a vicious cycle creating an environment favorable for the development of neurodegenerative diseases.** Such a relationship between these processes is mainly characteristic of the elderly and is the most likely reason for the increased incidence and adversity of COVID-19 among the elderly.

In summary, impairment in number, function, and activation of cells involved in the immune response [53–55] and aging of hematopoietic stem cells [56] are major phenotypes of the immune system associated with immunosenescence (Figure 2). Ultimately, these changes lead to a process termed "inflammaging," where low-grade inflammation is present at an advanced age and is associated with a worsening of chronic progressive medical conditions, such as congestive heart failure [57], and the onset of age-related diseases involving the central nervous system (e.g., Alzheimer's disease) [58]. When the age-associated inflammation persists in the long-term, it may lead to oxidative stress in various tissues, while also triggering organelle dysfunction (e.g., mitochondrial and lysosomal), which could, in turn, increase the cell vulnerability to infection.

### Inflammaging: an ally of SARS-CoV-2

An age-related decline in cellular repair mechanisms causes accumulation of damage at genome and proteome levels. This can lead to systemic changes in the immune system and increase pro-inflammatory cytokine production (interferon, interleukin, etc.), resulting in inflammaging [57]. The increase in cytokine production originates from the tissue macrophages, which initiate and regulate the inflammation [59]. Macrophages may, therefore, play significant roles in inflammaging. Some of the cellular hallmarks of aging, such as deregulated nutrient signaling and mitochondrial dysfunction, are also implicated in inflammaging, thus promoting the inflammatory environment [60].

Macrophages are also affected by aging, characterized mainly by the reduced potential for phagocytosis, and a decline in the gut barrier function [61]. Alveolar macrophages (AM) maintain lung homeostasis and play an important role in the influenza infection [62]. In particular, aged AM have a reduced power to control lung damage during influenza infection. During the progress of aging, the number of AM is reduced, leading to a lowered ability for phagocytosis [63]. Previous studies have also shown a decline of innate immune receptor functions and a substantial increase in viral replication efficiency after influenza infection in aged or senescent cells [64]. While the detailed mechanisms remain to be further studied, a reduction of the interferon (IFN) response in senescent cells after viral infection may play an important role. Moreover, a significant decrease in percentages and numbers of CD8+ T cells specific for at least one of the dominant epitopes of the influenza virus (influenza A nucleoprotein, NP, epitope) is typical for aged mice [65].

Pro-inflammatory cytokines play an important role in aging processes. The activation and the high levels of inflammatory cytokines such as IL-1, IL-6, TNF, and IFN-gamma are linked with morbidity and mortality in older patients [66]. In particular, IL-6 is a multifunctional cytokine produced in response to tissue damage and infections by multiple cell types [67]. Previous studies demonstrate its critical role in promoting lung tissue inflammation [68] and stimulating viral replication [69]. Moreover, elevated IL-6 is correlated with respiratory failure [10], and high concentrations of IL-6 in the serum is considered one of the hallmarks of severe MERS-CoV infections [70]. Additionally, an increase of IL-6 levels predicts adverse outcomes of COVID-19, underscoring inflammaging as the main ally of SARS-CoV-2 [35, 71]. Moreover, a recent study investigated the occurrence of cytokine storm in COVID-19 patients, also focusing on immunological characteristics of the response to COVID-19. In both mild and severe cases of COVID-19, increased levels of IL-6 are typical, while this is not the case among asymptomatic patients [10].

Inflammaging is also consistent with the gender bias of SARS-CoV-2. The more robust age-dependent activation of the innate pro-inflammatory pathways in COVID-19 is demonstrated in men compared to women [51], which is consistent with a higher rate of inflammaging among men [72]. A different situation among centenarians lends further support to the inflammaging importance for COVID-19 progression. Distinct longevity traits characterize centenarians, anti-inflammatory markers being the most prominent example, likely protecting them against the adverse outcomes of sustained inflammation as well as from the most severe forms of COVID-19 [73, 74].

Another critical factor is the impact of senescence in the lungs. Although COVID-19 shows symptoms across the entire body, the most prominent symptoms are respiratory and those associated with respiratory illness. The lung function tends to decrease with age having decreased alveolar elasticity [75], and increased senescence of epithelial cells and fibroblasts render cells frail to injuries such as the one caused by age-associated inflammation and viral infection [76]. Resident immune cells, most notably neutrophils, are also present in the lungs and are subject to immunosenescence. These cells become less functional due to age-associated chronic exposure to inflammatory cytokines [77], ultimately leading to fibrosis and aberrant tissue regeneration. The senescence phenotype, however, can be controlled by external factors, such as smoking [78], thus increasing the pool variability found in patients from the same age. In summary, the literature reviewed above may hold the key as to why the combination of immunosenescence and inflammaging does not allow an efficient response to the invasion of SARS-CoV-2 and why older individuals with co-morbidity are more prone to adverse outcomes of COVID-19 [79].

Diminished immune functions characterize immunosenescence, and inflammaging leads to a lack of anti-inflammatory modulators. The existing evidence suggests that inflammaging and immunosenescence, taken together, have vital roles in the decline of immune system functions to fight SARS-CoV-2 infection and lead to severe COVID-19 in older subjects (Figure 2).

### SARS-CoV-2: a possible tipping point for inflammaging and neurodegeneration

Aging is the most significant risk factor for the development of neurodegenerative diseases such as Parkinson's disease (PD), Alzheimer's disease, or amyotrophic lateral sclerosis (ALS). In PD, inflammation in the central nervous system (CNS), i.e., neuroinflammation, plays a vital role in the severity of the pathogenesis and is considered a key player in nigral cell loss [80].

Neuroinflammation is mainly regulated by glial cells, such as microglia and astrocytes. Microglia are considered the resident macrophages of the brain, therefore representing the first line of immune defense in the CNS. Moreover, they perform clearance of the metabolic waste, damaged cells, and pathogens, thus regulating both the pro-inflammatory and anti-inflammatory response [81]. During pathogenesis, microglia become activated due to cellular damage and the presence of protein aggregates in their surroundings, triggering the production of chemokines and cytokines such as TNF-α, IL-6, IL-1β, IFN-γ and CCL2 [82]. The resulting oxidative stress amplifies the damage to cellular components and further activates neighboring glial cells, thus causing a chronic activation [83]. Moreover, recent studies show that microglia can play a crucial role in defense of olfactory neuronal cells against viral infection [84]. Although data regarding the role of chemokines in SARS-CoV-2 infection is still scarce, it is known that infected epithelial cells upregulate genes encoding multiple chemokines such as CXCL1, CXCL3, CXCL6, CXCL16, and CXCL1. This increases the immune activation and recruitment of immune cells to the infected tissue, thus representing a potential therapeutic target [85].

It has been long established that peripheral inflammation associated with chronic diseases increases the production of cytokines, in particular IL-1β, in the CNS [86]. However, viral infections, such as with H1N1, can cause microglial activation [87]. This, in turn, increases the risk of developing diseases such as PD [88] and may trigger protein aggregation [89]. Another pointer towards neuro-immune crosstalk in neurodegeneration is the fact that nonsteroidal anti-inflammatory drugs also show a protective effect in the case of neurodegenerative diseases [90].

A milestone in the research on mechanisms of neuro-immune crosstalk was the discovery of the brain meningeal lymphatic system that clears proteins and metabolic waste from the cerebrospinal fluid (CSF) [91]. During aging, the lymphatic system becomes impaired due to a reduction in the lymphatic vessel diameter and leads to an increase in waste accumulation in the brain [92]. Such CNS-derived antigens contribute to the neuroinflammatory conditions, and their clearance is essential to counter the inflammation [91]. It is possible that due to peripheral inflammation, not only blood-borne cytokines can enter the brain, causing the detrimental neuroinflammatory effects, but also the immune cells present in the lymphatic system, exposing the brain to a vicious circle increasing its vulnerability to additional injuries.

The available literature on SARS-CoV-2 suggests that the virus may enter the nervous system via the lymphatic circulation [93]. SARS-CoV-2 can infect lymph endothelial cells [94] and, therefore, may use the paranasal lymph vessels to reach the brain. The presence of the virus was confirmed in the neuronal and capillary cells in the frontal lobe of the COVID-19 patients [95], associated with a worsening of neurological symptoms. The convergence of viral load in the nervous system and its relationship with brain lymphatics and microglial reaction against the virus may explain why some patients have prominent neurological symptoms, while others do not appear to experience these at all.

Aging triggers debilitating conditions, such as systemic low-grade inflammation and neurodegeneration. Such conditions can be set off or aggravated by viral infections, as evidenced by the H1N1 infection shown to contribute to PD development. The severity of SARS-CoV-2 infection indicates not only an overwhelming response of the immune system, but the presence of neurological symptoms suggests the connection with the CNS.

Severe neurological symptoms associated with COVID-19 have become increasingly noticeable after SARS-CoV-2 has been detected in the CSF of some patients [24]. A growing number of cases show neurological manifestations in COVID-19 patients, including examples of cerebrovascular disease, Guillain-Barré syndrome, encephalitis, and necrotizing encephalopathy [96]. The neurological symptoms appear in proportion with the severity of SARS-CoV-2 infection: patients with severe cases of COVID-19 show neurological manifestations (45.5%) with a higher incidence relative to the mild cases [97, 98]. The overall number of patients who displayed neurological symptoms is still low compared to respiratory manifestations. Still, the continuing pandemic and the data collected so far predict an increase in the number of neurological diseases that should not be underestimated [98]. It has also been proposed that SARS-CoV-2 infection may disrupt cellular homeostasis, ultimately leading to protein misfolding and, this way, increasing the propensity for the future development of neurodegenerative diseases [99].

This relationship calls for caution and extensive research related to the development of neuroinflammation and neurodegenerative diseases among COVID-19 survivors.

## CONCLUDING REMARKS

Our understanding of COVID-19 is growing by the day due to the increasing amount of clinical data and laboratory studies. The most prominent symptoms are associated with the tissues expressing the ACE2 receptor (airway epithelia and lung parenchyma). Still, the presence of neurological symptoms draws attention to the potential interaction of COVID-19 with the CNS.

Older people and people with co-morbidities are more prone to display severe symptoms of COVID-19 due to cellular senescence in the affected tissues and the immune system. Therefore, in the elderly, SARS-CoV-2 'preys' on the tissue debility and the deficiency of the immune system. The knowledge of immunosenescence and inflammaging provides a potential interpretation of epidemiological data underscoring the elderly as the population most sensitive to COVID-19.

Peripheral inflammation associated with aging and chronic diseases increases the production of cytokines also in the CNS. Similar effects can be triggered by viral infection via microglia activation, promoting protein aggregation, and, in turn, increasing the risk of developing neurodegenerative diseases [99]. Therefore, understanding the triangle between SARS-CoV2, immunosenescence, and inflammaging may shed important light on the molecular underpinnings of COVID-19, and open novel avenues for therapeutic interventions. These are desperately needed so that our lives can return to the 'normality' we used to know before this pandemic.
